# Functional Insights into *SlNPF*, *SlNRT2*, and *SlAMT* Gene Families in Tomato: Leaf Metabolic Performance Controls Root-to-Shoot Nitrogen Partitioning

**DOI:** 10.3390/plants14233642

**Published:** 2025-11-29

**Authors:** Juan Pablo Ledesma-Valladolid, Mayra Isabel Niño-González, Guadalupe Xóchitl Malda-Barrera, Ángel Ramón Flores-Sosa, Juan Ramiro Pacheco-Aguilar, Gerardo Manuel Nava-Morales, Edmundo Mateo Mercado-Silva

**Affiliations:** 1 Departamento de Investigación y Posgrado, Facultad de Química, Universidad Autónoma de Querétaro, Cerro de las Campanas S/N, Querétaro 76010, Mexico; jpledesma95@gmail.com (J.P.L.-V.); mayraa.2795@gmail.com (M.I.N.-G.); angel_ramon08@hotmail.com (Á.R.F.-S.); juanramiro29@yahoo.com.mx (J.R.P.-A.); 2 Facultad de Ciencias Naturales, Universidad Autónoma de Querétaro, Av. de las Ciencias S/N, Delegación, Juriquilla, Querétaro 76230, Mexico

**Keywords:** Nitrogen use efficiency (NUE), NPF transporter family, root-to-shoot signaling, nitrogen assimilation, gene expression analysis, bioinformatic analysis

## Abstract

Low Nitrogen Use Efficiency (NUE) remains a critical agricultural challenge, as an estimated 50–70% of applied nitrogen (N) is lost, resulting in negative environmental impacts and reduced crop production. To elucidate molecular mechanism controlling NUE in tomato (*Solanum lycopersicum*), we conducted a comprehensive genomic, transcriptomic, and functional analysis of the NPF, NRT2, and AMT transporter families under high-N commercial supply conditions. Our integrated analysis identified a shoot-to-root signaling mechanism where the plant’s metabolic performance systematically regulates N transport capacity. Under N sufficiency, the shoot exhibited reduced N assimilation, evidenced by NO_3_^−^ accumulation (increased by 55.7%) and reduced Nitrate Reductase (NR) and Glutamine Synthetase (GS) activities (54.0% and 43.2% reduction, respectively), which correlated with a 42.3% reduction in chlorophyll synthesis capacity. This reduction in metabolic demand systematically triggered the downregulation of the key long-distance *SlNPF transporter*s, *SlNPF2.13* and *SlNPF7.3*, restricting N translocation and promoting significant root N accumulation (increased by 41.8%). Our data established that the leaf metabolic state is the systemic regulator of N transport and identified *SlNPF2.13* and *SlNPF7.3* as pivotal molecular checkpoints. These findings indicate that the manipulation of these transporters could serve as a valuable tool in molecular breeding programs to significantly enhance NUE in commercial tomato varieties.

## 1. Introduction

Nitrogen (N) is the most critical macronutrient for plant growth and development, playing a vital role in synthesizing essential compounds such as amino acids, hormones, and chlorophylls [1,2,3,4,5,6]. Efficient N management is crucial for global agricultural productivity. Unfortunately, the high reliance on N-based fertilizers is linked to Low Nitrogen Use Efficiency (NUE), resulting in an estimated 50–70% of the applied nitrogen being lost during plant development [5,7,8]. These losses are detrimental to the environment, causing nutrient leaching, promoting the release of nitrous oxide (N_2_O), a potent greenhouse gas, and contributing to a 15–20% reduction in crop yield [8,9]. These challenges underscore the urgent need to enhance NUE for sustainable agriculture.

Tomato (*Solanum lycopersicum*) is one of the world’s most important horticultural crops, with an estimated global production value exceeding $70 billion USD annually [10]. Poor NUE severely impacts tomato yield, directly affecting profit margins. To achieve NUE optimization in this crop, it is crucial to understand the molecular bases of N uptake and internal partitioning. Nitrates (NO_3_^−^) and ammonium (NH_4_^+^) are the main inorganic forms of N taken up by plants [11,12] via specific membrane transport systems classified as high-affinity (HATS) and low-affinity (LATS). LATS is primarily composed of the nitrate transporter 1/small peptide transporter (NPF) family, enabling NO_3_^−^ uptake under high N availability (>0.5 mM). HATS includes the NRT2 family for NO_3_^−^ uptake and the AMT families (AMT1 and AMT2) for NH_4_^+^ acquisition, which operate primarily under low N conditions (˂0.1 mM and ˂1 mM for NO_3_^−^ and NH_4_^+^ acquisition, respectively) [6,11,12,13,14,15,16,17,18,19].

These transporter families are recognized as potential targets for improving NUE. However, precise knowledge gaps remain regarding their genomic annotation, evolutionary relationships, and, most importantly, the complex mechanism by which the plant’s shoot system metabolically regulates root N uptake and translocation (shoot-to-root signaling) [20]. Crucially, existing genomic annotations for these families in tomato present discrepancies; while some reports identified only four SlNRT2 genes [21], our comprehensive in silico analysis found six members, highlighting the need for an updated characterization. In tomato, previous studies have provided initial insights, such as the association of high N availability with reduced transcript levels of LATS (*SlNPF1.2*, *SlNPF2.6*, *SlNPF2.11*, *SlNPF7.1*, and *SlNPF7.3*) in both roots and leaves [22], and the downregulation of HATS (*SlNRT2.1* and *SlNRT2.3*) in roots [23]. Furthermore, while recent research supports the involvement of some members in uptake, transport, and stress tolerance [13,19,20,21], these studies often focus on individual gene responses and fail to provide an integrated mechanism linking the shoot’s metabolic status to the transcriptional regulation of these root-to-shoot transporters.

In this study, we conducted a comprehensive analysis of the *SlNPF*, *SlNRT2*, and *SlAMT* gene families in tomato. Our work integrates: (1) whole-genome characterization (evolutionary analysis, duplication, and structure); (2) transcriptomics; and (3) functional validation through metabolic analysis. We hypothesize that the metabolic state of the tomato shoot provides a systemic signal that transcriptionally regulates *SlNPF* long-distance transporters to restrict nitrogen translocation under high N sufficiency. Our results provide fundamental insights into the mechanism of systemic regulation of N transport, identifying *SlNPF2.13* and *SlNPF7.3* as pivotal molecular checkpoints for enhancing NUE in commercial tomato production.

## 2. Results

### 2.1. Identification and Physicochemical Characterization of SlNPF, SlNRT2, and SlAMT Gene Families in Tomato

A comprehensive BLASP search, using AtNPF (53 members), AtNRT2 (7 members), and AtAMT (5 members) protein sequences from *A. thaliana* as reference, led to the identification of 29 SlNPF, 6 SlNRT2, and 4 SlAMT members in the *Solanum lycopersicum* genome. We systematically evaluated the physicochemical characteristics of these transporter families, including coding sequence (CDS) length, protein length, molecular weight (MW), isoelectric point (pI), and predicted subcellular localization (Supplementary Table S1).

The *SlNPF* family exhibited the largest gene size; *SlNPF6.1* had the longest CDS length, measuring 1962 bp. In comparison, the maximum CDS lengths for the *SlNRT2* (*SlNRT2.1/SlNRT2.2*) and *SlAMT* (*SlAMT1.2*) families were shorter, measuring 1593 bp and 1545 bp, respectively. Overall, SlNPF proteins were the largest, with lengths ranging from 537 aa (SlNPF5.8) to 654 aa (SlNPF6.1), corresponding to MW values up to 71.92 kDa (SlNPF6.1). SlNRT2 and SlAMT proteins were smaller, ranging from 458 to 531 aa, which aligned with lower MW values. The SlNPF family also exhibited the widest pI variation (5.79 to 9.37), suggesting high functional diversity. Subcellular localization predictions indicated that most proteins across all three families were located at the cell plasma membrane, chloroplast membrane, and vacuole, consistent with their known function as nutrient transporters (Supplementary Table S1).

### 2.2. Phylogenetic Analysis of NPF, NRT2 and AMT Proteins

**Figure 1:**
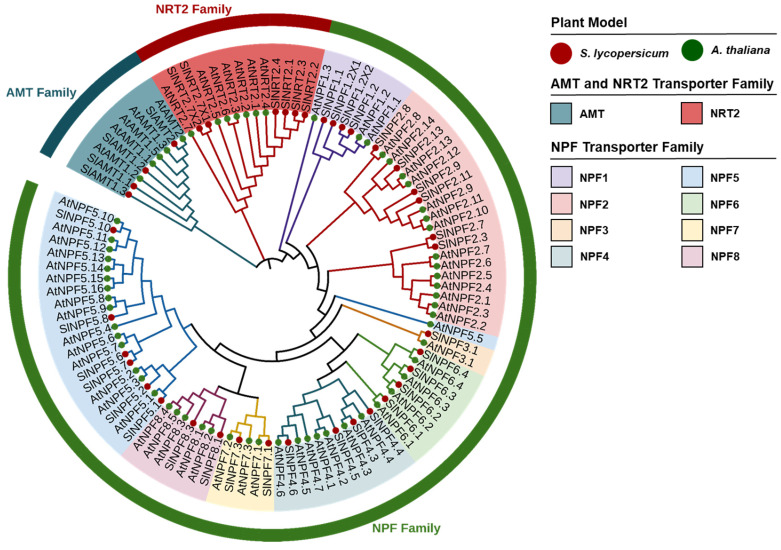
Phylogenetic relationships and evolutionary clustering of NPF, NRT2, and AMT protein families in *Solanum lycopersicum* (Sl) and *Arabidopsis thaliana* (At). The phylogenetic analysis was performed using the Neighbor-Joining method (MEGA XI software) with 1000 bootstrap replicates. The tree topology reveals ten distinct clusters, including a single highly conserved node for the AMT and NRT2 families, respectively. The NPF family is subdivided into eight subfamilies (NPF1–NPF8), each represented by a specific color.

We explored the interspecific and intraspecific homology, along with the evolutionary connections between SlNPF, SlNRT2, and SlAMT proteins by constructing a phylogenetic tree that included members from both *A. thaliana* and tomato. The resulting phylogenetic tree revealed a total of ten distinct clusters (Figure 1).

The AMT family showed high conservation, with all proteins from both species clustering into a single node (Figure 1). These SlAMT sequences exhibited a high mean homology of 78% between the two biological models. A similar clustering pattern emerged for the NRT2 family, where a single node encompassing all *A. thaliana* and tomato proteins was also identified (Figure 1). The NRT2 family proteins displayed high homology (67%) between the two species. This topology, characterized by the formation of single clusters for both AMT and NRT2 families, indicated a strong evolutionary conservation of these genes in tomato and *A. thaliana*.

In contrast, the NPF family proteins clustered into eight distinct subfamilies (NPF1-NPF8) (Figure 1). Unlike the patterns observed for the AMT and NRT2 families, the NPF subfamilies generally showed lower mean interspecific homology levels. The interspecific homology for each subfamily was as follows: 56.7% (NPF1), 44.6% (NPF2), 75.4% (NPF3), 41.5% (NPF4), 46.4% (NPF5), 52.1% (NPF6), 71.5% (NPF7), and 67.3% (NPF8) (Figure 1). The clustering of both species within specific NPF subfamilies further highlighted the shared ancestry and speciation events between them. Furthermore, the phylogenetic tree clearly showed the conformation of unique nodes for each transporter family, indicating distinct intraspecific gene duplication events within each model.

### 2.3. Analysis of Conserved Domain/Motif Analysis in SlNPF, SlNRT2 and SlAMT Proteins and Gene Structure Analysis of SlNPF, SlNRT2 and SlAMT Genes

**Figure 2:**
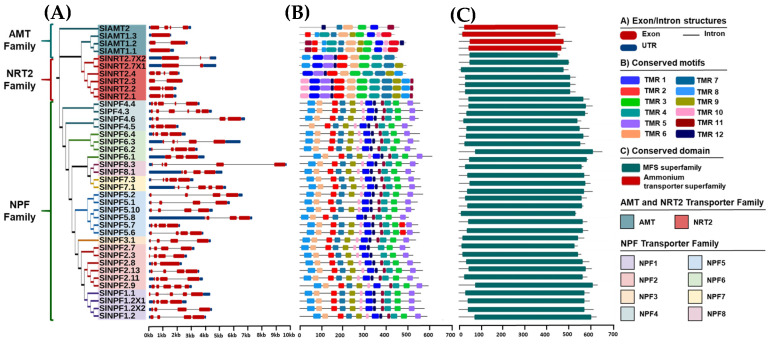
Structural analysis of *SlNPF*, *SlNRT2*, and *SlAMT* gene and protein families. (**A**) Exon/intron structure. Black lines represent introns, red boxes denote exons, and blue boxes denote UTR regions. (**B**) Conserved protein motif arrangements. (**C**) Conserved domain architecture. TMR, Transmembrane Region (corresponding to the conserved protein motifs).

We assessed the structural properties of the *SlAMT*, *SlNRT2*, and *SlNPF* genes and protein families to gain insights into their evolution in tomato. The gene structure analysis revealed wide divergence among the three families (Figure 2). The *SlAMT* family exhibited the least complex structure, with the *SlAMT1.3* and *SlAMT1.1* genes containing no introns (Figure 2A). Other *SlAMT* members showed between one (*SlAMT1.2*) and three (*SlAMT2*) introns, and one to four exons (*SlAMT1.1* and *SlAMT2*, respectively) (Figure 2A). In contrast, the *SlNRT2* family displayed a more complex structure, with the number of exons ranging from two to four, and introns from one to three (Figure 2A). The *SlNPF* family showed the highest structural divergence, with the number of exons ranging from three to seven (Figure 2A). Consistent with this high variability, 37.95% of the *SlNPF* genes contained five exons in their structure, suggesting a high rate of intron/exon gain or loss throughout the evolution of this family.

Protein motif analysis using the MEME tool identified conserved motif sequences corresponding to Transmembrane Regions (TMRs), which were confirmed by InterPro in all three transporter families (Figure 2B). Domain analysis revealed that the SlNRT2 and SlNPF families conserved a single domain: the Major Facilitator Superfamily (MFS) (Figure 2C). Correspondingly, SlNPF and SlNRT2 proteins shared a similar TMR arrangement, generally consisting of up to twelve conserved regions (Figure 2B). Specifically, SlNPF proteins showed slight variation, ranging from 10 TMRs (SlNPF5.8) to 12 TMRs (e.g., SlNPF6.1) (Figure 2B). Similarly, in the SlNRT2 family, while members such as SlNRT2.1, SlNRT2.2, and SlNRT2.3 possessed 12 TMRs, truncated arrangements (9–11 TMRs) were observed in the remaining proteins, with SlNRT2.7X2 having the lowest number of TMRs (9 TMRs) (Figure 2B).

Finally, the SlAMT protein family exhibited the conservation of the Ammonium Transporter Superfamily domain (Figure 2C). SlAMT proteins contained 11, 12, and 10 TMRs (SlAMT1.1, SlAMT1.2, and SlAMT1.3, respectively), with SlAMT2 showing the most divergent array with only 6 TMRs (Figure 2C).

### 2.4. Chromosome Distribution and Evolutionary Duplication Analysis of SlNPF, SlNRT2 and SlAMT Genes in Tomato

**Figure 3:**
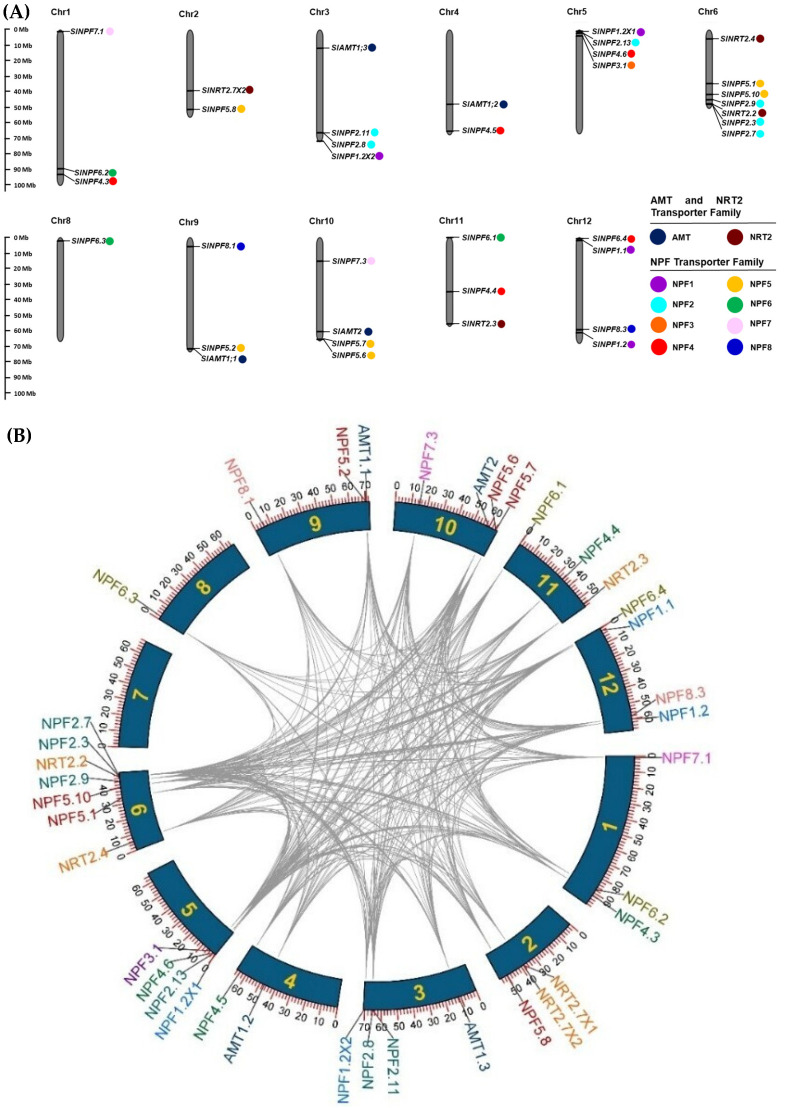
Chromosomal distribution and gene duplication analysis of the *SlNPF*, *SlNRT2*, and *SlAMT* gene families. (**A**) Distribution of the 39 identified genes across the 12 *Solanum lycopersicum* chromosomes. Chromosome lengths are shown in Mb (millions of bases). Gene subfamilies are specified by the same color. (**B**) Analysis of intraspecific gene duplication events (*S. lycopersicum*/*S. lycopersicum*). Segmental duplication pairs are linked by gray lines.

The 39 identified genes (*SlAMT*: 4, *SlNRT2*: 6, and *SlNPF*: 29) were disproportionately distributed across the 12 chromosomes of tomato (Figure 3A). Chromosome 6 contained the highest proportion of genes (29% of the total), while chromosome 7 contained no mapped genes (Figure 3A). To assess the evolutionary forces driving family size and distribution, gene duplication events were analyzed using the MCScan tool. Selective pressure was evaluated by calculating the non-synonymous (*Ka*) to synonymous (*Ks*) substitution ratio (Supplementary Table S2).

The NPF family exhibited a high number of duplication events (285 total) (Figure 3B). Segmental gene duplication events (258 pairs) were the dominant mechanism, significantly exceeding tandem duplication events (27 pairs) (Supplementary Table S2). All duplicated *SlNPF* genes pairs showed a *Ka*/*Ks* ratio ˂1 providing direct evidence of purifying selection during their evolution, suggesting the functional conservation of the encoded proteins (Supplementary Table S2).

For *SlNRT2* and *SlAMT* families, a lower number of intraspecific duplication events were detected (4 and 3, respectively) (Figure 3B). Segmental duplication was the dominant driving force in the *SlAMT* family, and its duplicated pairs also showed *Ka*/*Ks* ratios ˂1 (purifying selection) (Supplementary Table S2). The *SlNRT2* family evolution was influenced equally by both tandem and segmental duplication patterns. Only the *SlNRT2.7* X1/*SlNRT2.7* X2 gene pair had a *Ka*/*Ks* ratio >1, suggesting that diversifying selection played a secondary role in the evolution of *SlNRT2* family (Supplementary Table S2).

### 2.5. Expression Profiles of SlNPF, SlNRT2, and SlAMT Genes Under Nitrogen Deficiency Conditions

**Figure 4:**
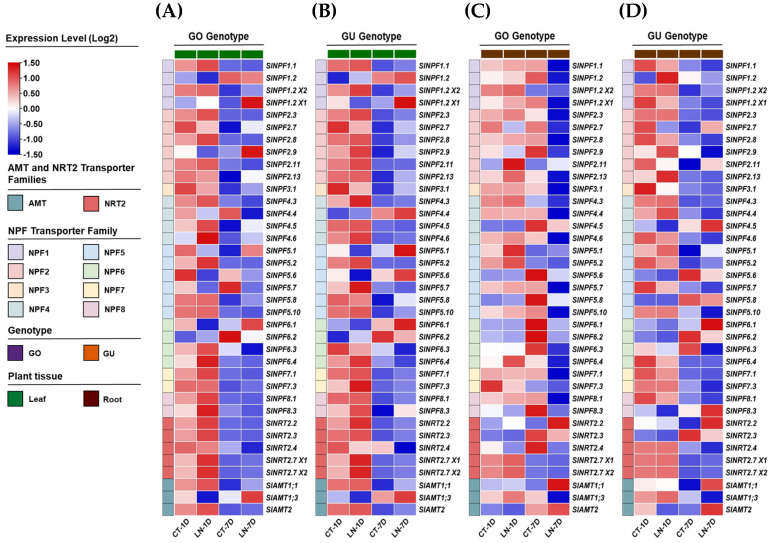
Exploratory RNA-seq analysis of *SlNPF*, *SlNRT2*, and *SlAMT* gene expression profiles in tomato (GO and GU genotypes) under nitrogen availability treatments. (**A**–**D**) Heatmap distribution showing the effect of nitrogen availability (Low nitrogen supply (LN) and optimal nitrogen supply (CT)) and exposure time (1 and 7 days) on gene expression in root and leaf tissues. FPKM values were normalized using *SlACT-41* as the internal control and transformed to Log_2_. The color scale indicates low (blue) to high (red) gene expression. Statistical analysis was performed via one-way ANOVA followed by Tukey’s test (*p* ≤ 0.05). Each treatment included three biological replicates.

An analysis of RNA-seq data from GEO-NCBI public database (bioProject accession number PRJNA578768) was conducted to provide an initial, broad characterization of the *SlNPF*, *SlNRT2*, and *SlAMT* family expression profiles. The data set included leaf and root samples from two tomato genotypes (GO and GU) subjected to low nitrogen (LN) and optimal nitrogen (Control treatment (CT)) supply for 1 and 7 days. This exploratory analysis revealed that nitrogen availability, exposure time, and plant genotype significantly affected gene expression (Figure 4A–D).

**Figure 5:**
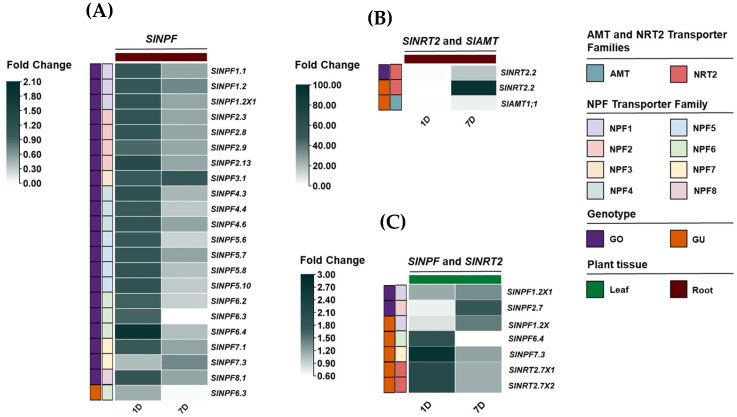
Heatmap visualization of differential gene expression (fold change) for *SlNPF*, *SlNRT2*, and *SlAMT* members that responded significantly to nitrogen deficiency. Heatmaps show the expression profiles (LN relative to CT) in root and leaf tissues of GO and GU genotypes at 1 and 7 days (*p* ≤ 0.05). (**A**) *SlNPF* family expression in root tissue. (**B**) *SlNRT2* and *SlAMT* family expression in root tissue. (**C**) *SlNPF* and *SlNRT2* family expression in leaf tissue. Statistical significance was determined by ANOVA followed by Tukey’s test (*p* ≤ 0.05).

A subsequent statistical analysis identified genes whose expression patterns responded significantly to nitrogen availability. Heatmaps were constructed with all significantly responding genes in the root and leaf tissues of the GO and GU genotypes, representing the fold change in gene expression under the LN condition relative to the CT condition on days 1 and 7 (Figure 5A–C and Supplementary Table S3).

The analysis revealed distinct expression patterns among the three transporter families. The high-affinity *SlNRT2* and *SlAMT* genes showed a clear trend of upregulation in both root and leaf tissues under LN conditions compared to CT (Figure 5B,C). In root tissues, the *SlNRT2.2* gene exhibited a notable increase in expression level on day 7, with transcript levels rising up to a 95.7-fold change, which highlighted its critical role in nitrogen uptake under N deficiency. In contrast, a general decrease in expression was observed on day 1. However, these changes were not statistically significant (Figure 5B). For leaf tissues, both *SlNRT2.7X1* and *SlNRT2.7X2* showed 2.1-fold increases in expression on day 1 (for each one) (Figure 5C). Conversely, a decrease was observed on day 7, though these changes were not statistically significant (Figure 5C).

Conversely, the low-affinity *SlNPF* genes exhibited a differentiated response between root and leaf systems. Under the LN condition, these genes were downregulated in root tissues on day 7, showing 0.008- to 0.693-fold changes (Figure 5A). This downregulation was most notable in GO genotype. The data showed a general increase in expression on day 1, but these changes were not statistically significant (Figure 5A). Interestingly, in leaf tissues, these low-affinity *SlNPF transporter*s were upregulated under LN condition (Figure 5C). For the GO genotype, *SlNPF1.2X1* and *SlNPF2.7* increased their expressions on day 7, showing 1.348- and 1.753-fold changes, respectively (Figure 5C). The changes observed on day 1 were not statistically significant. For GU genotype, *SlNPF1.2X1* increased its expression on day 7 (1.464-fold change), while *SlNPF6.4* and *SlNPF7.3* increased their expression on day 1, showing 1.905- and 2.751-fold changes, respectively (Figure 5C).

Based on these differential expression patterns, a subset of genes with a significant response to LN relative to CT was selected for further analysis in our experimental system, which included two conditions: N1, with higher ammonium availability (11.4 mM NO_3_^−^ and 2.3 mM NH_4_^+^), and N2, with lower ammonium availability (10.5 mM NO_3_^−^ and 0.5 mM NH_4_^+^). The specific genes selected for our study are listed in Supplementary Table S4.

### 2.6. Gene Expression Profile Analysis of SlNPF, SlNRT2, and SlAMT Families in Tomato

We performed functional validation of selected *SlNPF*, *SlNRT2*, and *SlAMT* genes (listed in Supplementary Table S4) by quantifying their expression levels via qRT-PCR in our experimental model under N1 and N2 conditions. This validation focused on a subset of genes that showed a significant response in the RNA-seq analysis.

#### 2.6.1. Gene Expression Profile Analysis

**Figure 6:**
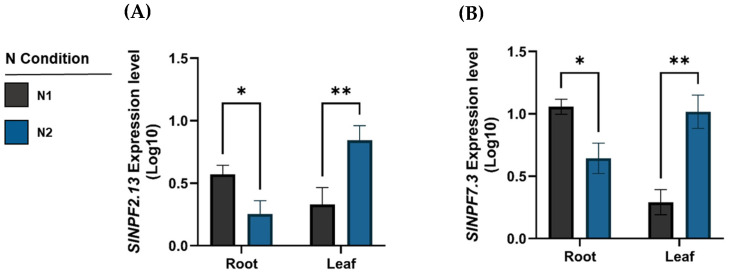
Relative expression patterns of the key *SlNPF2.13* and *SlNPF7.3* genes, measured by qRT-PCR under two commercial high N conditions (N1 and N2) in root and leaf tissues. (**A**) Relative expression (Log_10_) of the *SlNPF2.13* gene in root and leaf tissues. (**B**) Relative expression (Log_10_) of the *SlNPF7.3* gene in root and leaf tissues. All bar graphs represent the mean ± Standard Error (SE) of three biological replicates (*n* = 3). Statistical significance between N1 and N2 treatments within each tissue was determined using Student’s *t*-test (*p* ≤ 0.05). Asterisks (*) denote a statistically significant difference; ** indicates a highly significant difference (*p* ≤ 0.01).

Our qRT-PCR results revealed differential expression patterns between root and leaf tissues under N1 and N2 conditions. However, successful gene expression quantification was only possible for *SlNPF2.13* and *SlNPF7.3* (Figure 6A,B). In root tissues, both *SlNPF2.13* and *SlNPF7.3* exhibited a reduced expression level under the N2 condition (0.48- and 0.39-fold change, respectively). Conversely, leaf tissues under N2 condition showed an opposite response, with the expression levels of *SlNPF2.13* and *SlNPF7.3* increasing up to 3.3- and 5.3-fold changes, respectively (Figure 6A,B).

The non-detection of *SlNRT2* and *SlAMT* gene expression via qRT-PCR was consistent with the known regulatory mechanism of high-affinity transporters. These genes are typically repressed by high ambient nitrogen signaling, and the N1 and N2 conditions used in our study (11.4 mM NO_3_^−^ to 10.5 mM NO_3_^−^) exceed the necessary low-threshold concentrations (˂0.1 mM NO_3_^−^ and ˂1 mM NH_4_^+^, respectively) required for their transcriptional activation [12,13]. Therefore, our analysis focuses exclusively on the functional role of the constitutive *SlNPF transporter*s, which are less sensitive to high external N concentrations.

#### 2.6.2. Integrated Analysis of Gene Expression and Physiological Response

To functionally validate the role of the expressed *SlNPF* genes, we assessed nitrogen metabolism in roots and leaves, as well as chlorophyll synthesis capacity. Our findings revealed a differentiated metabolic performance between root and leaf samples, directly correlating with the observed *SlNPF2.13* and *SlNPF 7.3* expression patterns.

**Figure 7:**
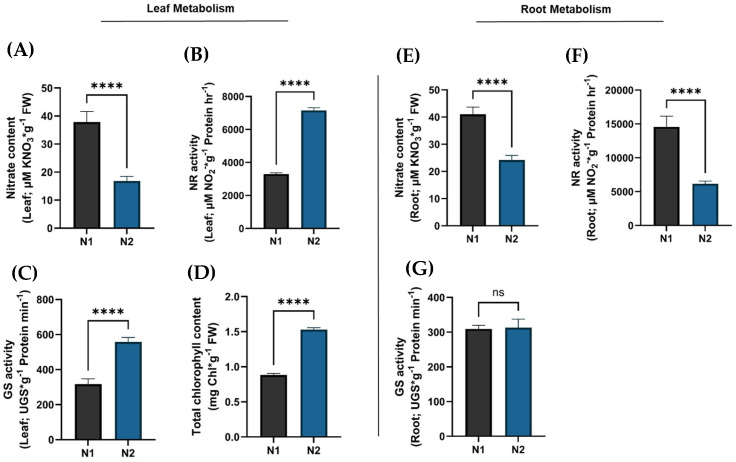
Physiological responses under two commercial high N conditions (N1 and N2) in leaf and root tissues. (N1 condition: gray bars; N2 condition: blue bars). (**A**) Nitrate content (µM KNO_3_ · g^−1^ FW) in leaf tissue. (**B**) Nitrate reductase (NR) activity (µM NO_2_^−^ · g^−1^ Protein · h^−1^) in leaf tissue. (**C**) Glutamine synthetase (GS) activity (UGS · g^−1^ Protein · min^−1^) in leaf tissue. (**D**) Total chlorophyll content (mg Chl · g^−1^ FW) in leaf tissue. (**E**) Nitrate content (µM KNO_3_ · g^−1^ FW) in root tissue. (**F**) Nitrate reductase (NR) activity (µM NO_2_^−^ · g^−1^ Protein · h^−1^) in root tissue. (**G**) Glutamine synthetase (GS) activity (UGS · g^−1^ Protein · min^−1^) in root tissue. All bar graphs represent the mean ± Standard Error (SE) of three biological replicates (*n* = 3). Statistical significance between N1 and N2 treatments within each tissue was determined using Student’s *t*-test (*p* ≤ 0.05). Asterisks (****) denote a highly statistically significant difference (*p* ≤ 0.0001); ns indicates no significant difference.

Leaf samples of plants subjected to the N1 condition exhibited a reduced metabolic capacity in comparison to those subjected to the N2 condition. NO_3_^−^ content was increased by 55.7% in N1 (Figure 7A). This high nitrate content was related to lower nitrate reductase (NR) and glutamine synthetase (GS) activity, showing a 54.0% and 43.2% reduction, respectively (Figure 7B,C). This suggested a lower conversion rate of nitrate to nitrite, indicating a reduced nitrogen assimilation capacity in leaves from N1 condition.

Furthermore, this reduced assimilation was associated with a 42.3% reduction in total chlorophyll content (Figure 7D), corroborating a reduced synthesis of intermediate metabolites necessary for chlorophyll synthesis. This lower metabolic capacity suggested a reduced nitrogen demand at the leaf level, which could explain the downregulation of *SlNPF2.13* and *SlNPF7.3* expression under N1 condition, thereby reducing nitrogen transport from roots to leaves. These responses, in turn, appeared to exert a strong regulatory control over root nitrogen uptake and metabolism, as described next.

**Figure 8:**
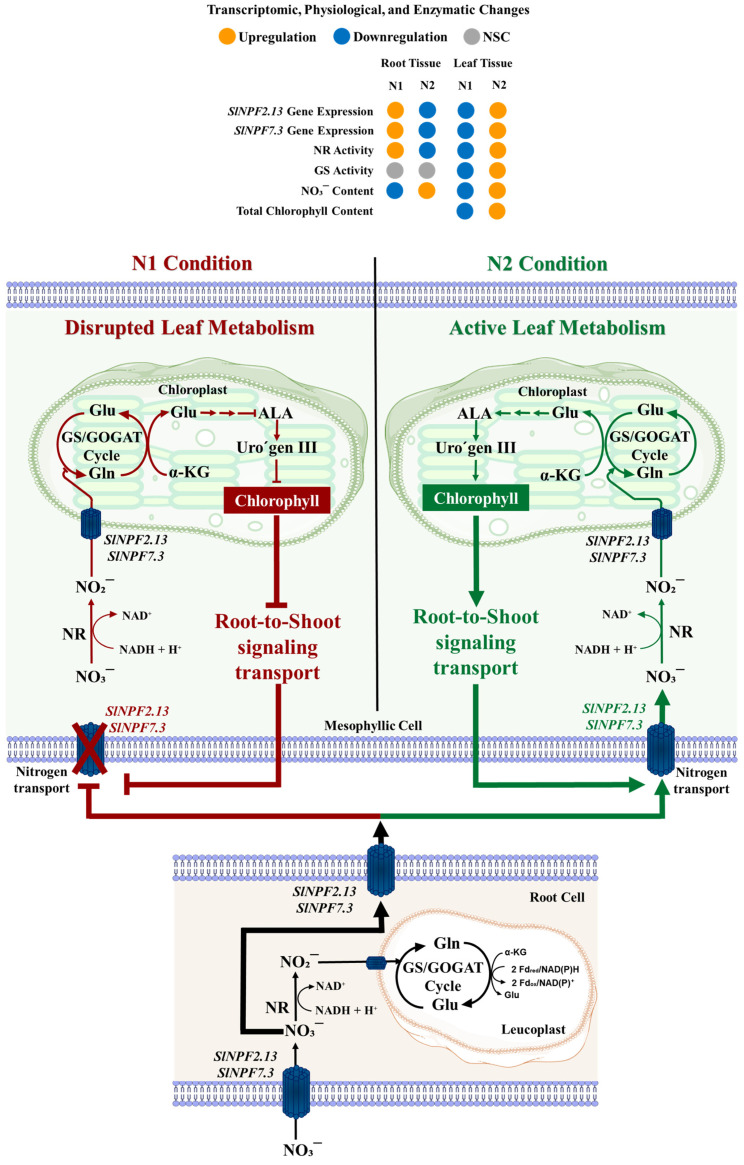
Proposed shoot-to-root signaling mechanism regulating nitrogen long-distance transport in tomato (*Solanum lycopersicum*). The scheme illustrates the control exerted by leaf metabolic performance over systemic N transport. Under the high-ammonium N1 condition (left side), leaf tissues exhibit a significantly reduced metabolic activity, as evidenced by high NO_3_^−^ accumulation and a concomitant decrease in NR/GS activities and chlorophyll content. This reduced N demand acts as a key systemic signal, which transcriptionally downregulates the *SlNPF transporter*s *SlNPF2.13* and *SlNPF7.3* in the shoot. This integrated response restricts long-distance N transport, leading to NO_3_^−^ accumulation in the root tissues, despite the local upregulation of these transporters at the root level. Conversely, the N2 condition (right side), which supports higher leaf metabolic activity, generates a greater N demand that stimulates N transport toward the shoot. The heatmap within the figure summarizes the qualitative changes observed for the gene expression levels (*SlNPF2.13* and *SlNPF7.3*), enzymatic activity (NR and GS), and metabolite content (NO_3_^−^ and chlorophyll) under N1 and N2 conditions: orange indicates high levels or upregulation, while blue indicates low levels or downregulation. The scheme was created using some graphic components adapted from Servier Medfical Art licenced under Creative Commons Attribution (CC BY 4.0) (Servier Medfical Art: (https://smart.servier.com/)).

Conversely, root of plants grown under the N1 condition showed a significant increase in nitrogen uptake and metabolism capacity compared to the N2 condition. NO_3_^−^ content was increased by 41.8% (Figure 7E). This higher metabolite concentration induced a 57.7% increase in NR activity (Figure 7F). GS activity did not show significant differences between N1 and N2 conditions (Figure 7G). While this enhanced uptake is partly driven by the higher N availability in the N1 solution, the higher expression level of both *SlNPF2.13* and *SlNPF7.3* in roots further facilitated this increased capacity. Critically, these root results, coupled with the reduced assimilation observed in leaves under N1 condition, suggested a severe restriction in nitrogen transport capacity from roots to leaves, resulting in the nitrogen metabolites accumulation within the root tissues (Figure 8).

## 3. Discussion

### 3.1. In Silico Identification and Evolutionary Insights

The initial bioinformatics analysis identified 29 *SlNPF*, 6 *SlNRT2*, and 4 *SlAMT* genes in the tomato genome (*Solanum lycopersicum*). The identification of six *SlNRT2* members contrasts with previous reports, such as the four members documented by [21]. This discrepancy is justified by the utilization of the most recent genomic assembly, Slycopersicum_390_v2.5, which refines the annotation of previously incomplete sequences. Furthermore, the earlier study omits quantitative cutoff criteria (such as the E-value used in BLASTP) (as of 1 September 2024; https://blast.ncbi.nlm.nih.gov/), which likely resulted in a more restrictive gene exclusion.

The size of these transporters families correlates consistently with the plant’s ploidy level [24]. For instance, the hexaploid *Triticum aestivum* exhibits 211 *TaNPF* members [25]. This pattern is consistent across allotetraploid plants such as *G. hirsutum* (98 members), *B. napus* (169 members), and *N. tabacum* (143 members). In contrast, diploid models, including tomato (29 members identified here), contain a lower number of *SlNPF* genes [18,24,26]. This count is comparable with other diploid species: *Z. mays* (78 members), *G. raimondii* (52 members), *G. arboreum* (51 members), *O. sativa* (96 members), *M. domestica* (73 members), and *S. oleracea* (57 members) [18,27,28,29,30]. A similar trend is evident for the *SlNRT2* family; diploid species such as tomato (6 members identified in this study), *Z. mays* (7 members), and *S. oleracea* (9 members) displaying a significant lower count compared to the hexaploid *T. aestivum* (49 members) [27,29,31]. These genomic comparisons establish tomato as a highly conserved diploid model concerning its nitrogen transporter families.

Analysis of the gene and protein sequences of these families offers insights into their evolutionary history. Gene structure analysis reveals that the *SlAMT1* family exhibits the lowest number of intron in its genomic sequence (*SlAMT1.1* and *SlAMT1.3* have none, and *SlAMT1.2* has only one). Since intron loss rates are generally faster than intron gain following gene duplication events [12,32], this simpler structure suggests the *SlAMT* family is evolutionary younger than the *SlNRT2* and *SlNPF* families. Furthermore, the *SlAMT* family may have evolved from the *SlNRT2* and *SlNPF* families. This hypothesis is supported by our gene duplication analysis, which detects 51 and 14 duplication events linking *SlNPF* and *SlNRT2* families to the *SlAMT1* family, respectively.

Furthermore, protein structural analysis confirms the presence of the conserved Major Facilitator Superfamily (MFS) domain across these families. The MFS domain, one of the oldest and most diverse superfamily of secondary transporters on Earth, is characterized by a conserved structure typically involving up to 12 transmembrane regions (TMRs) [33,34,35]. The extensive conservation of the *SlNPF* and *SlNRT2* families, maintained through numerous gene duplication events, strongly suggests that the evolution of these families is driven by purifying selection pressure (evidenced by the *Ka*/*Ks* ratio) to preserve the essential biological function inherent in the MFS domain.

### 3.2. Systemic Regulation: Shoot-to-Root Restriction Under N-Sufficiency

The integrated analysis provides strong evidence for a mechanism of nitrogen long-distance transport restriction tightly controlled by shoot-to-root signaling (Figure 8). The experimental design isolates the effects of the ammonium-to-nitrate ratio, where N1 condition provides moderate ammonium excess (an imbalance) versus N2 (optimal supply). The downregulation of *SlNPF2.13* and *SlNPF7.3* represents the key molecular checkpoints in this systemic regulation. Crucially, the lack of expression observed for *SlNRT2* and *SlAMT* (HATS) under both N1 and N2 conditions serves as an internal control, confirming that the plants were grown under high-N sufficiency, validating the focus on the *SlNPF* low-affinity system [12,13]. Under N1 condition, leaf tissues exhibit a reduced metabolic performance, evidenced by lower chlorophyll synthesis and reduced nitrogen assimilation capacity (lower NR and GS activity), signaling a reduced nitrogen demand at the shoot level. This reduction, in turn, triggers the observed downregulation of the root-to-shoot transporters *SlNPF2.13* and *SlNPF7.3*. This transport restriction explains the subsequent NO_3_^−^ accumulation and enhanced NR activity observed in the root, evidencing the root’s attempt to compensate for the shoot limitation. Furthermore, root metabolism capacity was triggered by the higher nitrogen availability in the nutrient solution in N1 condition, leading to the *SlNPF2.13* and *SlNPF7.3* upregulation (Figure 8).

### 3.3. Novel Metabolic Contrasts in Ammonium Toxicity

Despite extensive efforts to elucidate regulatory mechanisms under ammonium toxicity, the overall process remains unclear. Our findings provide key insights by linking the systemic signal to leaf metabolic status, highlighting a severe disruption of the optimal polysaccharide metabolism → glycolysis → tricarboxylic acid cycle → oxidative phosphorylation/mitochondrial metabolism flux [36,37,38,39].

Ammonium excess triggers this leaf metabolic disruption, which, in turn, regulates the photosynthetic capacity upstream. This regulation directly impacts chlorophyll synthesis and its critical relationship with nitrogen metabolism. Literature frequently suggests that ammonium toxicity causes an increase in chlorophyll synthesis capacity [40,41,42], potentially compensating for reduced photosynthetic efficiency and photosystem assembly capacity [36,43,44]. These responses are correlated with an increased nitrogen assimilation, which is generally understood to serve as an ammonium sink, avoiding the negative ammonium toxicity effects [36,37].

In stark contrast, our study evidences a reduced chlorophyll synthesis capacity and, consequently, a reduced nitrogen assimilation capacity at the leaf level. This differential response represents a critical molecular distinction in the mechanism of ammonium toxicity regulation. Evidence suggests a mutual regulatory mechanism exists between the chlorophyll synthesis pathway and nitrogen assimilation, mediated by Uroporphyrinogen III methyl transferase (UPM1) [45]. The reduced chlorophyll synthesis capacity observed under N1 condition triggers a subsequent reduction of nitrogen assimilation, effectively minimizing the nitrogen demand and initiating the disruptive shoot-to-root signaling pathway (Figure 8). Our work, therefore, proposes a novel protection-by-limitation mechanism, where toxic effects are minimized by limiting nitrogen transport, contrasting with the literature’s proposed mechanism of avoidance through increased assimilation sink capacity.

### 3.4. Functional Clarification and Implications for NUE Improvement

Our results provide strong evidence for long-distance transport, giving insights into the ongoing functional controversy surrounding both *SlNPF2.13* and *SlNPF7.3*. While NPF members such as *AtNPF2.12*, *AtNPF4.6*, and *AtNPF7.1* are known for their role in N redistribution [46,47], the specific function of *SlNPF2.13* and *SlNPF7.3* is widely debated. For instance, *SlNPF7.3* has been associated with K^+^ uptake [20], even as other evidence supports its involvement in NO_3_^−^ sensing and transport to aerial organs [22]. Similarly, although *SlNPF2.13* was historically linked to NO_3_^−^ remobilization [48], recent findings suggest a role as an H^+^/TM symporter involved in microbiome-plant interactions [49]. Taken together, our integrated analysis supports their primary involvement in NO_3_^−^ uptake and long-distance partitioning. The ability of these transporters to integrate systemic N-status signals confirms their pivotal role as key molecular checkpoints in the systemic regulation of NUE. The identification of these two genes mediating shoot-to-root restriction provides a clear roadmap for NUE optimization using both breeding and biotechnology strategies: specifically, sequence variations (SNPs or haplotypes) within the coding and promoter regions of *SlNPF2.13* and *SlNPF7.3* are promising candidates for Molecular Assisted Selection (MAS) [50]. This breeding strategy is further supported by polymorphism modification studies involving *OsNPF5.16* and *AtNRT1.1/AtNPF6.4*, which enhanced their expression patterns, promoting better growth and nutrient use efficiency [51,52]. Furthermore, biotechnology techniques such as CRISPR/Cas9 [53] can be used to modify promoter elements to fine-tune the degree of downregulation, potentially allowing the root to sustain higher NO_3_^−^ uptake even when the shoot experiences moderate NH_4_^+^ excess. Future research should focus on knockdown or CRISPR/Cas9 approaches targeting these two genes to definitively prove their role in this systemic signaling mechanism and determine their full potential for enhancing NUE in commercial tomato production.

## 4. Materials and Methods

### 4.1. Identification and Characterization of SlNPF, SlNRT2, and SlAMT Families in Tomato

To identify the *SNPF*, *SNRT2*, and *SlAMT* gene families in tomato, reference protein sequences from *Arabidopsis thaliana* were retrieved from the TAIR (The Arabidopsis Information Resource (TAIR) database: as of 1 August 2024; https://www.arabidopsis.org) and GenBank-NCBI (NCBI GenBank database: as of 1 August 2024, https://www.ncbi.nlm.nih.gov/genbank) databases [12]. These *A. thaliana* protein sequences served as the query set for a BLASTP search analysis against the whole protein sequences collection (CDS translations + PDB + SwissProt + PIR + PRF) archived in the GenBank (801,977,703 sequences; as of 1 September 2024) of *Solanum lycopersicum* (taxid: 4081). Crucially, the search was constrained to the *S. lycopersicum* genome assembly Slycopersicum_390_v2.5 (Phytozome v12). The cut-off values applied were: 50% identity, 90% coverage, and E-value threshold of 1 × 10^−10^. The accession numbers of the best proteins sequence hits were recorded and the corresponding protein, CDS, and genomic sequences were retrieved from the GenBank database. Data regarding gene and protein sequence length, intron and exon number, and gene chromosome location for both *A. thaliana* and tomato were retrieved directly from the NCBI database. Protein sequences were further subjected to physicochemical characterization: molecular weight (MW), isoelectrical point (pI), and protein length were analyzed using the Expasy ProtParam tool (Expasy ProtParam tool: as of 1 October 2024, https://web.expasy.org/protparam/). Subcellular localization analyses were performed using WoLF PSORT (WoLF PSORT prediction tool: as of 1 October 2024, https://www.genscript.com/wolf-psort.html) and Cell-PLoc 2.0 (Cell-PLoc 2.0 prediction tool: as of 1 October 2024, http://www.csbio.sjtu.edu.cn/bioinf/Cell-PLoc-2/) [18,25].

### 4.2. Multiple Sequence Alignment and Phylogenetic Analysis

To identify the interspecific and intraspecific homology, as well as to understand the evolutionary connections between SlNPF, SlNRT2, and SlAMT proteins in both, *A. thaliana* and tomato, a multiple sequence alignment analysis using the full-length amino acid sequences was conducted. This was performed using the ClustalW program with default alignment parameters [54]. Subsequently, an evolutionary relationships analysis was performed by constructing a neighbor-joining phylogenetic tree using the MEGA XI software. The tree construction utilized the Jones-Taylor-Thornton (JTT) model with 1000 bootstrap replications [18,31,55]. The high-quality phylogenetic tree map was generated using the iTOL online tool (Online tool iTol: as of 1 October 2025, https://itol.embl.de/).

### 4.3. Structure, Conserved Domain, and Motif Analysis of the SlNPF, SlNRT2, and SlAMT Families

The exon and intron structures of the *SlNPF*, *SlNRT2*, and *SlAMT* genes were analyzed using the Gene Structure Display Server (GSDS) (Gene Structure Display Server (GSDS): as of 1 October 2024, https://gsds.gao-lab.org/) online tool. Additionally, conserved protein domains were identified using the online web tools InterPro (Online server InterPro: as of 1 October 2024, https://www.ebi.ac.uk/interpro/) and The Conserved Domain Database (CDD) (NCBI Conserved Domain Database: as of 1 October 2024, https://www.ncbi.nlm.nih.gov/Structure/cdd/wrpsb.cgi) with an E-value threshold ˂0.01. Finally, the twelve most conserved protein motifs were identified using the MEME web tool (Web tool MEME for conserved protein motifs identification: as of 1 October 2024, https://meme-suite.org/meme/tools/meme), configured to identify 12 conserved motifs using default parameters [25,56,57]. The TBtools software (Software TBtools: as of 1 September 2024, https://github.com/CJ-Chen/TBtools-II) (v2.096) was used to visualize the conserved protein domains and motifs.

### 4.4. Chromosomal Distribution Analysis and Gene Duplication Analysis

The chromosomal locations of the *SlNPF*, *SlNRT2,* and *SlAMT* genes were determined using the web tool MG2C (Online tool MG2C for chromosome mapping: as of 1 October 2024, http://mg2c.iask.in/mg2c_v2.1/) (v2.1), utilizing physical chromosome location and length retrieved from NCBI. Gene duplication analysis for tomato was carried out through the MCScanX algorithm, using the software TBtools [25]. For all duplicated gene pairs, the nucleotide substitution parameters *Ka* (synonymous substitution rate) and *Ks* (non-synonymous substitution rate) were calculated. For this purpose, the corresponding CDSs were aligned using ClustalW (in MegaXI), and the *Ka*, *Ks*, and the *Ka*/*Ks* ratio were assessed using the Nei-Gojobori method (with the Jukes–Cantor substitution model) in Mega XI.

### 4.5. In Silico Analysis of Gene Expression Responses to Nitrogen Deficiency

Gene expression profiles of the *SlNPF*, *SlNRT2*, and *SlAMT* families under nitrogen deficiency conditions were analyzed using publicly available RNA-seq data. The expression values, reported as Fragments Per Kilobase per Million mapped fragments (FPKM), were retrieved from the GEO-NCBI dataset ([58] bioProject accession number PRJNA578768) (RNA-seq dataset from GEO-NCBI: as of 1 November 2024, https://www.ncbi.nlm.nih.gov/geo/query/acc.cgi?acc=GSE139405). The data set included leaf and root samples from two tomato genotypes (GO and GU) subjected to low nitrogen (LN) and optimal nitrogen (Control treatment-CT) supply for 1 and 7 days. The FPKM values of target genes were normalized against the FPKM value of the reference gene *SlACT-41* for each sample to estimate relative expression. All relative expression values were subsequently subjected to log_10_ transformation. A heat map was constructed using the software TBtools (v2.096).

### 4.6. Plant Materials and Treatments

The present study was conducted in two commercial greenhouses under soilless culture systems (SCSs) from April 2022 to February 2023. Both greenhouses are located in Queretaro, Mexico (20°42′22.5′′ N 99°56′27.6′′ W). They are of the gothic type, equipped with polyethylene roofs and side walls for passive ventilation, and are oriented from north to south. The total area of the greenhouses was 5000 m^2^ and 7500 m^2^ for N1 and N2 treatments, respectively. After 16 days of germination, seedlings of cultivar “Merlice” were grafted onto the commercial rootstock “Maxifort”. This grafting practice is standard in commercial soilless systems to enhance root vigor and ensure high plant development uniformity across the plots. Two weeks later, the grafted plants were transplanted into commercial soilless grow bags (110 cm × 20 cm × 12 cm), which contained sterile coconut fiber as the growing substrate.

A drop irrigation system was used to supply nutrient solutions based on the Hoagland formula. The experiment consisted of two treatments: N1 (moderate ammonium excess, NH_4_^+^: NO_3_^−^ proportion of ~20%) and N2 (control with an optimal NH_4_^+^: NO_3_^−^ proportion of ~5%). This optimal range (5% to 10%) is recommended for tomato in soilless culture [59]. Average electrical conductivity and pH were 2.9 mS cm^−1^ and 6.1 in the N1 treatment, and 1.9 mS cm^−1^ and 6.4 in the N2 treatment. Average day/night temperature, relative humidity, and radiation for both N1 and N2 treatments were 20.9/12.3 °C, 63.2/77.8% and 16,176 J cm^−2^, respectively.

### 4.7. Sample Collection

Root (*n* = 15 per time point) and leaf (*n* = 15 per time point) samples were collected from plants grown under both N1 and N2 conditions at two distinct time points along the crop development: week 4 AST and week 8 AST. Root samples were collected ~20 cm away from the sprout union and shaken to remove substrate particles. The sixth fully expanded compound leaf was collected, considering the top compound leaf the first one. All individual samples collected at weeks 4 AST (*n* = 15) and 8 AST (*n* = 15) were pooled and subsequently clustered to form the final 3 composite biological replicates for each tissue (root and leaf) and treatment (N1 and N2). Each final composite biological replicate consisted of 10 individual samples, specifically comprising 5 samples from week 4 AST and 5 samples from week 8 AST. Once the biological replicates were prepared, they were placed in sterile sample bags and transported in a cooler (~4 °C) to the laboratory within 1.5 h of collection. Then, in the laboratory, they were immediately frozen with liquid nitrogen and stored at −80 °C.

### 4.8. Gene Expression Analysis (qRT-PCR)

Total RNA was extracted from frozen samples using the Plant/Fungi Total RNA Purification Kit (NORGEN Biotek Corp., Thorold, *ON*, Canada; Cat. 25800). gDNA residuals were removed by rigorous DNase treatment using the TURBO DNA-free^TM^ Kit (Thermo Fisher Scientific., Waltham, MA, USA; Cat. AM1907). First-strand cDNA was synthesized using the Maxima First Strand cDNA Synthesis Kit for RT-qPCR (Thermo Fisher Scientific., Waltham, MA, USA; Cat. K1641).

Gene expression quantification was performed via qRT-PCR using the PowerUp^TM^ SYBR^®^ Green Master Mix (Thermo Fisher Scientific., Waltham, MA, USA; Cat. A25742) on a CFX96^TM^ Real-Time System (BIO-RAD, Hercules, CA, USA). The qRT-PCR amplification conditions were as follows: UDG activation (one cycle at 50 °C for 2 min) and polymerase activation (one cycle at 95 °C for 2 min), followed by 40 cycles as follows: denaturation (95 °C for 10 s), annealing (59 °C for 30 s), and extension (72 °C for 30 s). A final dissociation curve (50 to 95 °C) was generated to verify the primer specificity.

Gene expression was quantified using the absolute method based on standard curves and normalized against the absolute expression of the internal control gene (*SlACT-41*). For final data representation, the relative expression values of each gene were further normalized against the lowest recorded expression value across all treatments and tissues to ensure positive values before log_10_ transformation. Each composite biological replicate (*n* = 3) was analyzed in technical duplicates.

For more methodology details, see the Supplemental Methodology, Section S1.1. Primer sequences for the target genes (*SlNPF*, *SlNRT2*, and *SlAMT*) and the internal reference gene, Actin (*SlACT-41*), are listed in Supplementary Table S4.

### 4.9. Physiological and Enzymatic Analysis

We carried out the assessment of nitrate content [60,61] in leaf and root tissue, Nitrate Reductase (NR) activity [62,63,64] in leaf and root tissue, Glutamine Synthetase (GS) activity in leaf and root tissue, protein content [63,65,66,67] in leaf and root tissue, and total Chlorophyll content [65,68] in leaf tissues. The detailed methodologies are described in Supplementary Methods, Section S1.2.

### 4.10. Statistical Analysis

All data are represented as the mean ± Standard Error (SE) of 3 biological replicates (n = 3). The statistical analysis of the public FPKM data (bioProject accession number PRJNA578768) was performed using the statistical package JMP 8. A two-way ANOVA was applied to the log_10_ transformed expression values to test the main effect of the nitrogen treatment (LN vs. CT), regardless of genotype, tissue, or days of exposure to those conditions. Only genes demonstrating significant response to nitrogen treatment (*p* ˂ 0.05) were selected as being nitrogen-responsive and the fold change in LN relative to CT was estimated. Experimental data from qRT-PCR, metabolite content, and enzyme activity were analyzed through the GraphPad Prism 10 package. Differences between N1 and N2 were evaluated within each tissue using Student’s *t*-test (*p* ˂ 0.05).

## 5. Conclusions

This study provides essential genomic and evolutionary insights for the *SlNPF*, *SlNRT2*, and *SlAMT* transporter families in tomato. Our work critically unveils a novel systemic mechanism, where the leaf metabolic status, characterized by reduced chlorophyll synthesis capacity under high ammonium availability, initiates a signal that regulates nitrogen transport. This signal triggers a protection-by-limitation mechanism by differentially downregulating the long-distance transporters *SlNPF2.13* and *SlNPF7.3*, resulting in NO_3_^−^ accumulation in the root and mitigating the negative effects of ammonium availability in the shoot.

This discovery moves beyond correlation by identifying these two *SlNPF* genes as key molecular checkpoints that control systemic nitrogen distribution. The practical relevance is significant. This knowledge offers a clear molecular roadmap for optimizing nitrogen fertilization programs and improving resource management in commercial tomato cultivation. Future research must experimentally validate this shoot-to-root mechanism using targeted approaches, specifically CRISPR/Cas9 or gene knockdown, to precisely fine-tune the expression patterns of *SlNPF2.13* and *SlNPF7.3* for enhanced NUE and sustainable crop production.

## Data Availability

The authors declare that all relevant data supporting the findings of this study are included in this article.

## Supplementary Materials

The following supporting information can be downloaded from https://www.mdpi.com/article/10.3390/plants14233642/s1, Table S1: Physicochemical properties of SlNPF, SlNRT2, and SlAMT families of *A. thaliana* and *Solanum lycopersicum*; Table S2: *Ka*/*Ks* values of *SlNPF*, *SlNRT2*, and *SlAMT* gene pairs duplicated genes in tomato; Table S3: Fold change values of *SlNPF*, *SlNRT2*, and *SlAMT* genes in root and leaf tissues with a significant response to LN condition relative to CT condition on days 1 and 7; Table S4: Sequences of the primer pairs used in the qRT-PCR analysis of the candidate genes.
