# Supplementary material for: Functional Insights into SlNPF, SlNRT2, and SlAMT Gene Families in Tomato: Leaf Metabolic Performance Controls Root-to-Shoot Nitrogen Partitioning
Source: Plants (Basel). 2025 Nov 29;14(23):3642. doi: 10.3390/plants14233642 (PMC12694162; doi:10.3390/plants14233642)
Supplement: Supplementary file 1 [file plants-14-03642-s001.zip › Supplementary_Methodology.pdf]

S1. Supplementary Methodology

S1.1. Gene expression analysis (qRT-PCR)

S1.1.1. RNA extraction, purification, and cDNA synthesis

Total RNA was extracted from the frozen samples of both root and leaf tissues using the Plant/Fungi Total RNA Purification Kit (NORGEN Biotek Crop. Cat. 25800), following the manufacturer´s guidelines. To maximize the RNA yield, the final elution step for RNA recovery was performed twice, as recommended by the manufacturer. RNA concentration and purity were subsequently verified using a NanoDrop spectrophotometer (260/280 nm). To remove gDNA residuals, the total RNA was treated with the TURBO DNA-free™ Kit (Thermo Fisher Scientific. Cat. AM1907), specifically applying the rigorous DNase treatment protocol as per manufacturer´s guidelines. First-strand complementary DNA (cDNA) was synthesized using the Maxima First Strand cDNA Synthesis Kit for RT-qPCR (Thermo Fisher Scientific. Cat. K1641), following the manufacturer´s instructions. RNA concentration and purity were assessed using a NanoDrop spectrophotometer (260/280 nm).

S1.1.2. Primer validation and gene expression quantification

Primer sequences for the target genes (*SlNPF*, *SlNRT2*, and *SlAMT*) and the internal reference gene, Actin (*SlACT-41*), are listed in **Supplementary Table S1**. Primer specificity and amplicon size were initially validated using standard RT-PCR. The reaction was performed using the Phire Green Hot Strat II DNA Polymerase (Thermo Fisher Scientific. Cat. F122S) using a C1000 Touch System, BIO-RAD. The PCR protocol was as follows: Initial denaturation (One cycle at 98 °C for 30 s), followed by 35 cycles as follows: denaturation (98 °C for 5 s), annealing (57 °C for 5 s), extension (72 °C for 15 s), and a final extension (One cycle at 72 °C for 1 min). PCR amplicons were examined on agarose gel (1.5% w/v) and visualized under UV light.

Gene expression quantification was performed via qRT-PCR using the PowerUp™ SYBR® Green Master Mix (Thermo Fisher Scientific. Cat. A25742) on a CFX96™ Real-Time System, BIO-RAD. The qRT-PCR amplification conditions were as follows: UDG activation (One cycle at 50 °C for 2 min) and polymerase activation (One cycle at 95 °C for 2 min), followed by 40 cycles as follows: denaturation (95 °C for 10 s), annealing (59 °C for 30 s), and extension (72 °C for 30 s). A final dissociation curve (50 to 95 °C) was generated to verify the primer specificity.

Gene expression was quantified using the absolute method based on standard curves, which were generated by plotting the Ct values against the cDNA concentration (ng) templete. The initial amplicon concentration (ng of amplicon) was obtained from the standard curves and used to estimate the number of copies (molecules) (absolute expression). Then, absolute expression was expressed as molecules per ng of total RNA. Relative expression was calculated by normalizing the absolute expression of the target genes to the absolute expression of the internal control gene (*SlACT-*

41). For final data representation, the relative expression values of each gene were further normalized against the lowest recorded expression value across all treatments and tissues to ensure positive values before log_{10} transformation.

S1.2. Physiological and enzymatic analysis

S1.2.1. Nitrate content assay in leaf and root tissue

For nitrate content assessment, 0.125 g frozen samples were extracted with 6 mL of 10 mM potassium phosphate buffer (pH 8.2) containing 10 mM EDTA and 5 mM cysteine. The mixture was homogenized with an Ultra-Turrax (IKA, T-25D) at 10,000 rpm for 1 minute and centrifuged (5,100 rpm for 15 min). The reaction mixture consisted of 100 µL filtrate and 200 µL 5% salicylic acid in concentrated H_2SO_4. After 20 min of cooling at room temperature, 9.5 mL 2 M NaOH was added to raise the pH to 12. After cooling the reaction mixture at room temperature, the absorbance was measured at 410 nm. Nitrate content was measured using a calibration curve with KNO_3 as a standard. The results were expressed in µM KNO_3 * g^{-1} FW [1,2].

S1.2.2. Nitrate Reductase activity assay in leaf and root tissue

For nitrate reductase activity (NR) assessment, 0.2 g frozen samples were extracted with 8 mL of 25 mM sodium phosphate buffer (pH 7.4) containing 50 mM cysteine and 1 mM EDTA. The mixture was homogenized with an Ultra-Turrax (IKA, T-25D) at 10,000 rpm for 1 minute and centrifuged (8,000 rpm for 10 min). The reaction mixture consisted of 400 µL filtrate and 1200 µL of 100 mM sodium phosphate buffer (pH 7.5) containing 100 mM KNO_3^- and 400 µL of NADH solution (2 mg mL^{-1}). After incubating at 30 °C for 30 min, 1 mL of 1% (w/v) p-aminobenzene sulfonic acid and 1 mL of 0.2% 1-Naftilamine were added. After cooling the mixture at room temperature for 20 min, the absorbance was recorded at 540 nm. The results were expressed in µM NO_2^- * g^{-1} Protein * h^{-1} [3–5].

S1.2.3. Glutamine synthetase activity assay in leaf and root tissue

For glutamine synthetase activity (GS) assessment, the extraction procedure was carried out as it was described in NR methodology. The reaction mixture consisted of 700 µL filtrate and 1600 µL of 100 mM Tris-HCl buffer (pH 7.4, 80 mM $MgSO_4$, 20 mM sodium glutamate, 20 mM cysteine, 2 mM EDTA, and 80 mM hydroxylamine hydrochloride) and 700 µL of 40 mM ATP solution. After incubating at 25 °C for 15 min, 1 mL of chromogenic reagent (0.2 M trichloroacetic acid, 0.37 M $FeCl_3$, 0.6 M HCl) was added. After incubating at 25 °C for 15 min, the reaction mixture was centrifuged at 5000 rpm for 10 min at 25 °C. Supernatant was collected, and the absorbance was measured at 540 nm. Reaction mixture without hydroxylamine hydrochloride was considered as the blank. The results were expressed in UGS * g^{-1} Protein * min^{-1}. Protein content was estimated by the Bradford method [4,6–8].

S1.2.4. Chlorophyll content assay in leaf tissue

For Chlorophyll content assessment, 0.2 g frozen samples were extracted with 25 mL of alcohol:acetone solution (v/v – 1:1). The mixture was homogenized with an Ultra-Turrax (IKA, T-25D) at 10,000 rpm for 1 minute and centrifuged (10,000 g for 1 min at 4 °C). The absorbance was measured at 663 and 645 nm using a UV-VIS spectrophotometer (PerkinElmer, UV/VIS Lambda 365) to estimate the total chlorophyll content expressed as mg Chl * g^{-1} FW [6,9].

References

1. Cataldo, D.A.; Maroon, M.; Schrader, L.E.; Youngs, V.L. Rapid Colorimetric Determination of Nitrate in Plant Tissue by Nitration of Salicylic Acid. Communications in Soil Science and Plant Analysis 1975, 6, 71–80, doi:10.1080/00103627509366547.
2. Yu, L.-H.; Wu, J.; Tang, H.; Yuan, Y.; Wang, S.-M.; Wang, Y.-P.; Zhu, Q.-S.; Li, S.-G.; Xiang, C.-B. Overexpression of Arabidopsis NLP7 Improves Plant Growth under Both Nitrogen-Limiting and -Sufficient Conditions by Enhancing Nitrogen and Carbon Assimilation. Sci Rep 2016, 6, 27795, doi:10.1038/srep27795.
3. Iqbal, A.; Dong, Q.; Wang, X.; Gui, H.; Zhang, H.; Zhang, X.; Song, M. Transcriptome Analysis Reveals Differences in Key Genes and Pathways Regulating Carbon and Nitrogen Metabolism in Cotton Genotypes under N Starvation and Resupply. IJMS 2020, 21, 1500, doi:10.3390/ijms21041500.
4. Iqbal, A.; Qiang, D.; Zhun, W.; Xiangru, W.; Huiping, G.; Hengheng, Z.; Nianchang, P.; Xiling, Z.; Meizhen, S. Growth and Nitrogen Metabolism Are Associated with Nitrogen-Use Efficiency in Cotton Genotypes. Plant Physiology and Biochemistry 2020, 149, 61–74, doi:10.1016/j.plaphy.2020.02.002.
5. Ren, B.; Dong, S.; Zhao, B.; Liu, P.; Zhang, J. Responses of Nitrogen Metabolism, Uptake and Translocation of Maize to Waterlogging at Different Growth Stages. Front. Plant Sci. 2017, 8, 1216, doi:10.3389/fpls.2017.01216.
6. Zhong, C.; Cao, X.; Hu, J.; Zhu, L.; Zhang, J.; Huang, J.; Jin, Q. Nitrogen Metabolism in Adaptation of Photosynthesis to Water Stress in Rice Grown under Different Nitrogen Levels. Front. Plant Sci. 2017, 8, 1079, doi:10.3389/fpls.2017.01079.
7. Wang, G.; Ding, G.; Li, L.; Cai, H.; Ye, X.; Zou, J.; Xu, F. Identification and Characterization of Improved Nitrogen Efficiency in Interspecific Hybridized New-Type Brassica Napus. Annals of Botany 2014, 114, 549–559, doi:10.1093/aob/mcu135.
8. Bradford, M.M. A Rapid and Sensitive Method for the Quantitation of Microgram Quantities of Protein Utilizing the Principle of Protein-Dye Binding. Analytical Biochemistry 1976, 72, 248–254, doi:10.1016/0003-2697(76)90527-3.
9. Wu, Y.; Li, Q.; Jin, R.; Chen, W.; Liu, X.; Kong, F.; Ke, Y.; Shi, H.; Yuan, J. Effect of Low-Nitrogen Stress on Photosynthesis and Chlorophyll Fluorescence Characteristics of Maize Cultivars with Different Low-Nitrogen Tolerances. Journal of Integrative Agriculture 2019, 18, 1246–1256, doi:10.1016/S2095-3119(18)62030-1.
